# Chimpanzee extractive foraging with excavating tools: Experimental modeling of the origins of human technology

**DOI:** 10.1371/journal.pone.0215644

**Published:** 2019-05-15

**Authors:** Alba Motes-Rodrigo, Parandis Majlesi, Travis Rayne Pickering, Matthias Laska, Helene Axelsen, Tanya C. Minchin, Claudio Tennie, R. Adriana Hernandez-Aguilar

**Affiliations:** 1 Department of Early Prehistory and Quaternary Ecology, University of Tübingen, Tübingen, Germany; 2 Centre for Ecological and Evolutionary Synthesis, Department of Biosciences, University of Oslo, Oslo, Norway; 3 Department of Anthropology, University of Wisconsin-Madison, Madison, WI, United States of America; 4 Plio-Pleistocene Palaeontology Section, Department of Vertebrates, Ditsong National Museum of Natural History (Transvaal Museum), Pretoria, South Africa; 5 Department of Physics, Chemistry and Biology (IFM), Linköping University, Linköping, Sweden; 6 Kristiansand Zoo, Kristiansand, Norway; Max Planck Institute for the Science of Human History, GERMANY

## Abstract

It is hypothesized that tool-assisted excavation of plant underground storage organs (USOs) played an adaptive role in hominin evolution and was also once considered a uniquely human behavior. Recent data indicate that savanna chimpanzees also use tools to excavate edible USOs. However, those chimpanzees remain largely unhabituated and we lack direct observations of this behavior in the wild. To fill this gap in our knowledge of hominoid USO extractive foraging, we conducted tool-mediated excavation experiments with captive chimpanzees naïve to this behavior. We presented the chimpanzees with the opportunity to use tools in order to excavate artificially-placed underground foods in their naturally forested outdoor enclosure. No guidance or demonstration was given to the chimpanzees at any time. The chimpanzees used tools spontaneously in order to excavate the underground foods. They exhibited six different tool use behaviors in the context of excavation: probe, perforate, dig, pound, enlarge and shovel. However, they still excavated manually more often than they did with tools. Chimpanzees were selective in their choice of tools that we provided, preferring longer tools for excavation. They also obtained their own tools mainly from naturally occurring vegetation and transported them to the excavation site. They reused some tools throughout the study. Our new data provide a direction for the study of variables relevant to modeling USO extractive foraging by early hominins.

## Introduction

It has been hypothesized that plant underground storage organs (USOs) were key dietary resources for African Pliocene and Pleistocene hominins who lived in dry and/or open environments [1–3]. Data on hominin craniodental anatomy [4–7], dental microwear [8–11], enamel chemistry [10, 12–16], and archaeology [17] are consistent with this hypothesis, indicating that USOs were likely to have been important in the diet of at least some species in the genera *Australopithecus*, *Paranthropus*, and *Homo*. Further, ecological studies on the abundance [18] as well as on mechanical properties of USOs [19] that grow in environments similar to those reconstructed for some hominin species, show that USOs were potentially an important food supply for hominins in such environments. In addition, ethnographic data from contemporary human hunter-gatherers (*Homo sapiens*) living in dry habitats demonstrate that USOs are staple foods in the diet of many of these populations [20–23]. However, despite this evidence, the answers to the questions of how USO extractive foraging developed in early hominins and how USOs could have played an adaptive role in hominin evolution remain elusive.

Some monkeys, including baboons (*Papio* spp.) [24], geladas (*Theropithecus gelada*) [25], Japanese monkeys (*Macaca fuscata*) [26] and black capuchins (*Sapajus nigritus*) [27] excavate shallow USOs by hand (usually <10 cm deep for baboons and geladas [2] and up to 16 cm deep for Japanese monkeys [26]). Wild chimpanzees (*Pan troglodytes*) at Tongo, Democratic Republic of Congo [28] and at Bossou, Republic of Guinea [29], have been observed to manually extract and consume USOs without the use of tools. There is both direct and indirect evidence that wild chimpanzees at Assirik, Senegal [30], also consume USOs, although no evidence that they extract these resources with tools has been reported. Indirect data from Ugalla, Tanzania, were the first to indicate that wild chimpanzees use tools to excavate edible USOs [31]. Sticks and pieces of bark, with wear inferred to be excavating use damage, were recovered from sites frequented by chimpanzees, and where different species of USOs had clearly been extracted and eaten [31]. More recently, Gaspersic and Pruetz [32] reported that wild chimpanzees from Bandafassi, Senegal, also use tools to excavate edible USOs, but did not provide detail about the evidence supporting this assertion. The only other non-human animals known to use tools to harvest USOs are bearded capuchins (*Sapajus libidinosus*) from Serra da Capivara National Park, Brazil, where they have directly been observed using stones to extract roots and tubers [33, 34].

The discovery that wild chimpanzees and bearded capuchins use tools to excavate USOs is important because this behavior was once considered unique to hominins, one which supposedly set humans and their ancient ancestors apart from other primates. The incorporation of USOs into the hominin diet was thought to have played a critical role in human evolution because these resources were proposed to have served as fallback foods in the transition from moister, forested to drier habitats, where USOs were more abundant [3]. Consequently, behavioral and anatomical adaptations to the consumption of USOs would have been important in facilitating the colonization of dry habitats by early hominins. In this context, the study of USO extractive foraging by nonhuman primates is particularly relevant to building and testing models of USO extractive foraging by our early hominin ancestors.

Chimpanzee USO extractive foraging may be especially important to such modeling as these apes are extremely closely related to humans, show a similar degree of encephalization and body size to early hominins, and include populations that reside in the kind of dry, open, seasonal environments reconstructed for many hominin species (see reviews in, e.g., [31, 35, 36]). Regarding the latter argument, the savanna chimpanzees of Ugalla and Bandafassi would be the ideal subjects for close study of the tool-assisted USO extractive foraging behavior of nonhuman hominoids. However, despite continuing evidence of USO consumption by Ugalla chimpanzees [37, 38], this population remains largely unhabituated to human observers and thus direct observations of USO excavating behavior are still lacking. Similarly, chimpanzees at Bandafassi are unhabituated and not directly observable. Thus, there is a gap in our knowledge regarding this extractive foraging behavior of potentially high relevance for human evolution. In order to fill this gap, we designed two experiments involving chimpanzees at the Kristiansand Zoo in Kristiansand, Norway. The chimpanzees, who were naïve to any activity in any context related to excavating, were presented with opportunities to extract artificially-placed underground foods in a naturally forested outdoor enclosure either manually or using tools, a task designed to simulate the excavation of USOs by wild chimpanzees.

Previous studies have presented two captive primate species with the task of excavating in order to obtain food. Tufted capuchins (*Sapajus apella*) were provided with sticks in order to extract soil-buried peanuts from containers [39]. In addition, two groups of captive bonobos (*Pan paniscus*) were provided with different tools to obtain food placed below two consecutive layers of condensed sand and stones in two distinct settings: one group was tested on an outdoor pit where stone, branch and antler tools were available and a second group was presented with a plastic box in their indoor quarters where only branch and antler tools were given [40]. The primates in all three experiments succeeded in extracting the buried food using tools. However, until now, no previous experimental study has ever presented chimpanzees, the only hominoid other than humans known to engage in tool-assisted USO extractive foraging in the wild, with the task of excavating in order to obtain underground food.

Our first experiment (Experiment 1) was a tool use and tool selection study. In this experiment we aimed to investigate i) whether and how the chimpanzees would use tools to excavate buried food, ii) if soil compactness would influence tool use excavation, and iii) if chimpanzees would be selective in their choice of tools. Since chimpanzees had never been observed excavating with tools, we were not able to make any hypotheses or predictions about our first aim. Regarding our second aim, we hypothesized that the compactness of the soil would affect the frequency in which the chimpanzees used tools for excavation. We predicted that they would use tools more frequently to excavate more compacted soil. In previous studies of tool selection, wild chimpanzees have been reported to select and reuse tools non-randomly based on tool characteristics such as size and weight [41–43]. Thus, regarding our third aim, we hypothesized that if the chimpanzees used tools for excavating underground food in our study, they would select tools based on physical characteristics (e.g. length, diameter, weight) that would be appropriate for excavation (i.e. large and heavy). Thus, we predicted that they would select larger and heavier tools from the ones experimentally provided, and that they would reuse some tools more frequently than others. Based on the tools reported to be used by chimpanzees in the wild for excavating USOs [31], we predicted that chimpanzees would use both sticks and bark as tools in our study.

Our second experiment (Experiment 2) was a study of excavating modalities (manual versus tool) and of tool selection. In this experiment we aimed i) to compare tool and manual excavation in terms of number of bouts and bout duration, and ii) to compare the physical characteristics of the excavating tools used by the chimpanzees when tools *were not* experimentally provided with those used by the chimpanzees in Experiment 1, when tools *were* experimentally provided. Regarding our first aim, we hypothesized that chimpanzees would excavate manually more often than using tools based on observations conducted during Experiment 1. We predicted that manual excavation would occur at higher frequencies as well as in longer bouts than tool excavation. Regarding our second aim, we hypothesized (based on our observations from Experiment 1) that when we did not experimentally provide tools in this second experiment the chimpanzees would obtain their own tools from the natural vegetation available in their enclosure in order to excavate and that these selected tools would have physical characteristics adequate for excavation. Thus, we predicted that the tools chimpanzees would obtain in Experiment 2 would be of similar physical characteristics to those of the tools they selected in Experiment 1, even if they would require more energy and time to obtain or make.

## Materials and methods

### Study subjects

**Fig 1:**
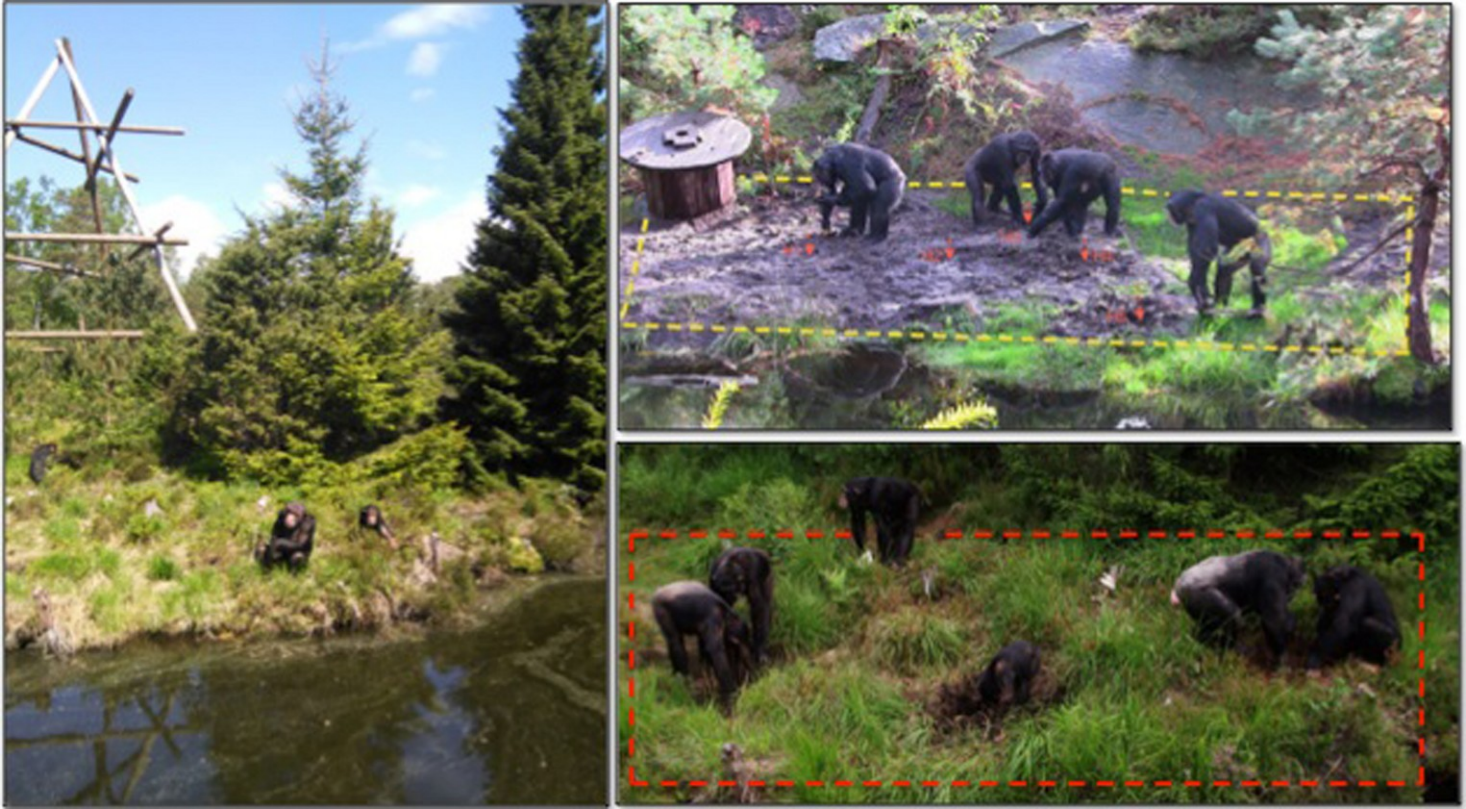
The outdoor enclosure or island. The yellow and red rectangles indicate the study areas during Experiments 1 and 2 respectively. The left picture illustrates the natural vegetation present in the island consisting of trees, shrubs, herbs and grasses.

Our study was conducted on a colony of chimpanzees (*Pan troglodytes*) housed at the Kristiansand Zoo in Kristiansand, Norway. All individuals included in the study were readily identified by the observers both live and from video recordings. During Experiment 1, the colony consisted of 10 individuals (four adult males, five adult females, and one two-year old infant female. See S1 Table for more details on demographics). During Experiment 2, the colony consisted of nine individuals (four adult males, four adult females and one five-year old juvenile female). The individuals were the same in both experiments, except for one adult female who had been relocated to another zoo prior to Experiment 2. All, except two individuals, were born in captivity (S1 Table). During the two experiments, the chimpanzees had access to two enclosures (one indoor and one outdoor), as well as to a separate indoor sleeping area. The indoor enclosure was equipped with climbing ropes, feeders and an artificial waterfall. In the indoor enclosure the chimpanzees had access to several enrichment devices: a tree trunk hanging from a chain with holes of different depths filled with honey that the chimpanzees obtained by using both their fingers and tools; an automatic dispenser that released nuts into a maze, which the chimpanzees could obtain by guiding the nuts with tools inserted through different holes; an artificial termite mount baited with honey for “fishing” with tools; and PVC tubes and hose fragments approximately 20cm long with honey smeared inside, which the chimpanzees obtained using tools and their fingers. The chimpanzees were also provided with long fresh branches (>1 m length) in the indoor enclosure. These branches retained side branches, leaves, and bark. The chimpanzees made tools by breaking the side branches and removing the leaves. The outdoor enclosure was an island of 1840 m^2^ surrounded by a water-filled moat, with natural soil, rocks and vegetation. The zoo is contained in a natural pinewood forest and the area that is now the island was made by digging a moat around a part of this forest. Thus, the vegetation within it is natural. At the time of the study the island had 35 trees (> 5 meters high) and plenty of shrubs, herbs and grasses that covered the vast majority of the island (Fig 1). In addition, the island had four wooden climbing frames and two small shelters. The indoor sleeping area was off-exhibit.

All chimpanzees participated voluntarily in this study. There were no changes to their feeding routines and they were never deprived of food or water. During Experiment 1, due to aggression from the alpha male towards the infant of the group, the chimpanzees were divided in two groups (N_group1_ = 5, N_group2_ = 5; S1 Table). This separation meant that each testing day one group was allowed onto the island while the other remained indoors. The two groups could not see each other while being in different enclosures. During Experiment 2 all chimpanzees were in a single group. Prior to this study all subjects were naïve to any activity related to excavating in any context. We confirmed this information by conducting interviews with all the chimpanzee keepers. The experiments reported here comply with the European Union Directive on the Protection of Animals Used for Scientific Purposes (EU Directive 2010/63/EU), the American Society of Primatologists’ Principles for the Ethical Treatment of Primates, and with current Norwegian and Swedish laws. The University of Oslo, Linköping University and the ethical board of the Kristiansand Zoo approved this study before its commencement.

### Experimental setting and design

#### Experiment 1

**Fig 2:**
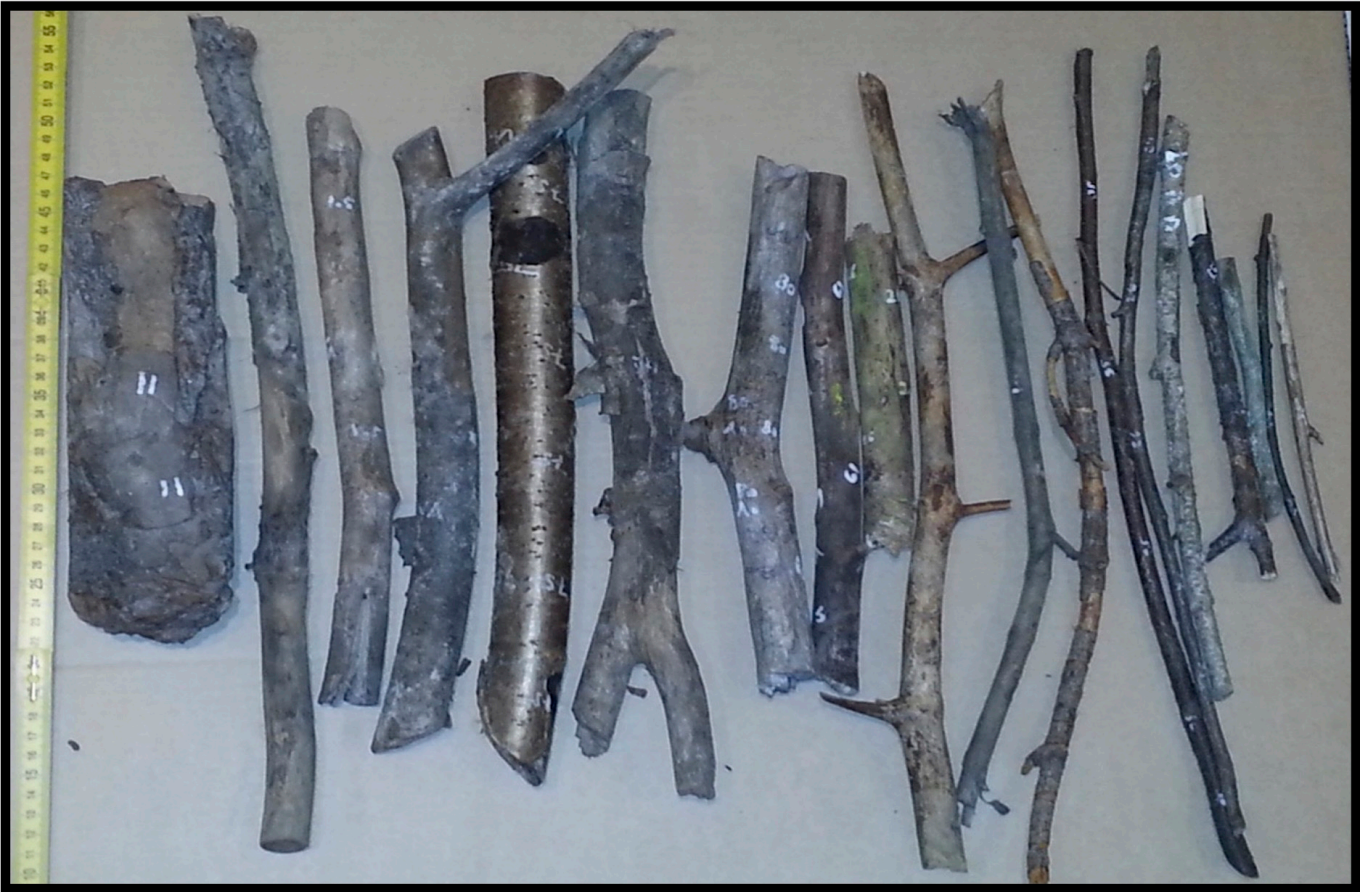
Sample of the tools provided to the chimpanzees (sticks and bark) in Experiment 1.

This experiment was a tool selection and use experiment. In this experiment we investigated the excavating behavior of the chimpanzees when they were provided with ready-made tools, which they could use for excavating. With this experiment we aimed to answer the following questions: i) would the chimpanzees use tools to excavate buried food?; ii) would the frequency of excavating tool use vary depending on the compactness of the soil?; iii) would they select excavating tools based on certain physical characteristics of these tools?; iv) would the chimpanzees reuse excavating tools?. Based on the tool materials that Hernandez-Aguilar et al. [31] found to be used by wild chimpanzees for USO extraction, the tools provided to the chimpanzees in our study were sticks from shrubs and trees, and pieces of bark from trees (Fig 2), obtained from a forest near Kristiansand. Except for the bark pieces (N = 14), the tools were sorted into categories according to their combined thickness (≤ 5, > 5 - ≤ 15, > 15 - ≤ 25, and > 25 mm) and length (≤ 20, > 20 - ≤ 40, and > 40 - ≤ 60 cm). Five to fifteen stick tools from each category were provided, except for the thickest and longest category (> 25 mm in thickness and > 40 - ≤ 60 cm in length) because the keepers wanted to minimize the risk if the chimpanzees would throw them (N = 110). These categories were chosen to provide the chimpanzees with tools of distinct dimensions that would have different leverage or strength. Together, we provided a total of 124 tools in Experiment 1. Each tool was marked with an ID number and its physical characteristics were recorded (length, weight, maximum and minimum end diameters). All tools were provided only once during the experiment, at the beginning of the *Loose Soil Condition* (see below). The tools were spread at approximate equal distance from each other in a linear formation, in a random order, within a length of three meters around the east side of the study area.

#### Experiment 1 included three conditions

Open Hole Condition: This was the initial condition of the study. We excavated five holes (15 cm in diameter and 30 cm in depth) in the study area. These dimensions were based on a field experiment of excavating USOs from the same species and size as those found to be obtained by wild chimpanzees in Issa, Tanzania (Hernandez-Aguilar, unpublished data). The holes were separated from one another by at least one meter (range 1–3 m). The holes remained open throughout this condition and each day one whole fruit (e.g. apple, nectarine, mango) was deposited into each hole in the morning before the chimpanzees were allowed onto the island. We did not conduct any control for food preferences but chose to use fruit because the keepers suggested the chimpanzees might like it better than all other food they get regularly (vegetables, primate pellets and nuts). This condition was included in order to familiarize the chimpanzees with the presence of food inside the holes excavated in the study area.

Loose Soil Condition: A whole fruit was placed at the bottom of each hole and the holes were filled with non-compacted soil from the island until each hole was filled to the ground level.

Compacted Clay Condition: A whole fruit was buried in each hole, but the holes were now filled to the ground level with grey clay instead of soil. We brought in the clay to the zoo from a forest close to Kristiansand and then used a hand tamper about ten times and our feet and body weight to compact the clay in the study area.

#### Experiment 2

This experiment was a study of the differences between excavating modalities (manual or tool), and the selection of tools in a more naturalistic setting than Experiment 1 (when no tools were provided to the chimpanzees and no additional substrates, such as clay, were added to the soil). In this case, the chimpanzees could only obtain their own tools from the natural vegetation of the island or from the fresh branches provided as enrichment in the indoor enclosure. Most of the trees on the island were pine trees, and consequently both dead and alive branches, sticks, and pinecones, were available. With this experiment we aimed to answer the following questions: i) would tool and manual excavation differ in terms of frequency (the number of bouts) and duration (bout duration)?; ii) would the physical characteristics of the excavating tools used by chimpanzees differ if tools *are not* experimentally provided compared to when tools *are* provided?.

#### Experiment 2 included two conditions

Open Hole Condition: Same as in Experiment 1. This condition was included in Experiment 2 to serve as a reminder of the experimental set up, as well as to attract the chimpanzees towards the new study area where Experiment 2 took place.

Compacted Soil Condition: A whole fruit was buried in each hole and the holes were filled with natural soil from the island to the ground level. We compacted the natural soil of the island manually and by foot approximately ten times per hole. Unlike in Experiment 1, in this experiment there was no *Loose Soil Condition* and no clay was used for the *Compacted Soil Condition*. The omission of the *Loose Soil Condition* was due to the different aim of this experiment compared to Experiment 1. In this experiment we did not explore how the compactness of the soil affected tool use excavating behavior, but rather how frequently (number of bouts) and for how long (bout duration) the two excavating modalities (manual and tool) were used. We did not use clay in this experiment because we aimed to create more naturalistic experimental conditions by not adding any substrate to the soil nor by providing tools to the chimpanzees.

For each experiment, a separate section of the island was selected as the study area (Fig 1). The selection of a different study area in each experiment was due to the clay remains present in the study area used in Experiment 1. As mentioned above, in Experiment 2 we aimed at creating more naturalistic conditions than in Experiment 1 in order to study the differences between excavating modalities (manual and tool). We observed the chimpanzees in both experiments from an observation point about five meters across the moat from the study areas. During both experiments the study area was prepared in the morning prior to the chimpanzees’ release onto the island (at about 8:00 a.m.). During the *Open Hole Condition* this preparation consisted in placing the fruits in the holes. During the remaining conditions every morning each hole was resized to its original dimensions (15 cm in diameter and 30 cm in depth) before placing the fruit and filling it with soil or clay. Each empty or filled hole was marked with a flag in an effort to potentially increase the strength of the association between the presence of food and the holes of the study area. The flag in Experiment 1 was made with a craft stick (12 cm in length, 0.3 cm wide, 2 g in weight) and in Experiment 2 with a bamboo skewer (30 cm in length, 0.3 cm wide and 2 g in weight) both with a yellow tag. The chimpanzees were unable to observe the study area during these preparations as they were in the indoor sleeping quarters. Once the chimpanzees were allowed onto the island, all had free access to the study areas and could voluntarily participate in the experiments. No guidance or demonstration was given to the chimpanzees at any time. To the best of our knowledge (and after consultation with the chimpanzee keepers), the chimpanzees had never observed another chimpanzee excavating nor a human excavating either manually or with tools. Therefore, they were naïve to the task we tested.

### Data collection

#### Experiment 1

Data collection was conducted from the 11^th^ of June to the 3^rd^ of October 2013 for a total of 66 days. The durations of the different conditions varied between the two chimpanzee groups as they were allowed a different number of days onto the island. The group that included the infant (group 2) was only allowed outdoors on sunny days to protect the infant's health. The *Open Hole Condition* lasted 16 days for group 1 and 7 days for group 2, the *Loose Soil Condition* lasted 11 days for group 1 and 8 days for group 2, and the *Compacted Clay Condition* lasted 17 days for group 1 and 7 for group 2. Sessions started between 8:00 and 9:00 a.m. and lasted approximately five hours (approximate total duration = 330 h). We used a digital video camera (Canon Legria HF M56) for recordings. We conducted video analysis using VLC Media Player Version 2.0.8 TwoFlower. Each different use of a tool in the context of excavating was defined as an excavating behavior (see S2 Table). A tool-excavating event was defined as the uninterrupted use of a tool during the performance of a single excavating behavior exhibited by an individual. A tool-excavating event ended when the chimpanzee changed the tool used, dropped the tool, moved to another hole, paused for more than three seconds [44] or performed a different excavating behavior. When the chimpanzees obtained their own tools from the natural vegetation of the island (“selected-non-provided tools”), these were measured, weighed, marked with ID numbers, and placed back where they were found by the experimenter. Data on tool reuse, tool transport, and tool modification by the chimpanzees were collected. Tool transport distances within and nearby the study area were estimated from video recordings. The straight distance between the holes and between holes and the corners of the study area and other natural features such as the moat line had been measured at the beginning of the study and these known distances were used to estimate each transport distance observed. When new tools were brought by the chimpanzees into the study area (“selected-non-provided tools”), the transport distance was obtained the following day by measuring with a tape the straight line from the place where the tool was obtained to the first hole where it was used. Tool modification was defined as a change in the physical properties of the object (sensu [45]) by the action of a chimpanzee. Every morning, the ID number of each tool used and not used the previous day was recorded and its position inside the study area was mapped (following [43]) before the chimpanzees were allowed to enter the island. We identified the individual tool that a chimpanzee was using from observations on-site and from video analysis.

#### Experiment 2

Data collection was conducted from the 24^th^ of May to the 30^th^ of September 2016 for a total of 78 days. The *Open Hole Condition* lasted 3 days and the *Compacted Soil Condition* lasted 75 days. The *Open Hole Condition* was shortened in this experiment compared to Experiment 1 because the chimpanzees already had the previous experience with the setup by the time of Experiment 2. Recordings lasted 30 minutes during the *Open Hole Condition* (total duration = 1.5 h) and approximately 3 hours during the *Compacted Soil Condition* (approximate total duration = 225 h), starting when the chimpanzees were allowed onto the island between 8:00 and 9:00 a.m. Sessions were recorded with a digital video camera (Canon Legria HF M56). We conducted video analysis using Quick time player version 10.4. The first aim of this experiment was to compare manual and tool excavation in terms of frequency and duration. An excavating bout was defined as the period of time during which an individual continuously excavated (following [46]), starting at the moment the chimpanzee introduced a hand or a tool in one of the holes and ending when the chimpanzee changed the tool used, dropped the tool, moved to another hole, paused for more than three seconds [44] or performed a different excavating behavior. Bouts were classified as tool use or manual bouts depending if a tool was used at any time during the bout or not, respectively. The chimpanzees obtained their own tools from the natural vegetation of the island or from the fresh branches provided as enrichment in the indoor enclosure. Every morning, the tools present in the study area that were associated to the holes and showed signs of being used the previous day by the chimpanzees (e.g. soil adhered to one or both ends of the tool) were permanently removed from the study area, weighted and measured.

### Data analysis

Statistical analyses were carried out using R version 2.8.0.

In order to investigate how chimpanzees used tools to excavate in Experiment 1, we performed a descriptive analysis in which we created an ethogram of the different excavating behaviors observed. We calculated the frequencies with which each excavating behavior occurred in each condition and how many observations of each excavating behavior were performed per individual.

In order to determine if the different compactness of the soil had an effect on the frequency of tool use in Experiment 1, we used a Generalized Linear Mixed Model [47] with Poisson error structure and log link function. We used as a response the number of tool use excavating events performed by each individual in each day of the two experimental conditions that involved soil filled holes. As test predictor we used the experimental condition (*Loose Soil* or *Compacted Soil*), which was a priori manually dummy coded, and we controlled for sex. We included subject as random effect and condition within subjects as random slope. When examining the model stability, we found that overdispersion was high (4.3), indicating that the response deviates more from the model than expected given a Poisson distribution because too many values deviate from the mean. Consequently, we proceeded using instead a negative binomial model using the function glmer.nb of the R package lme4 with the same model structure. This new model led to a much less overdispersion value of 1.3. No collinearity issues were found (function vif of the R package car; [48]). The significance of the model was established comparing the full model with a reduced null model in which the test predictor "condition" was not included using a likelihood ratio test [49] with the test Chisq in the R function *anova*. The number of days each individual was tested was not included as an offset term as the response was already computed per day.

In order to compare tool and manual excavation in Experiment 2, we used the two-tailed Wilcoxon signed rank test for paired samples to compare the total duration of bouts, total number of bouts, the daily mean bout durations and the daily mean number of bouts of manual and tool excavation of the individuals who excavated using both modalities. We decided to calculate the individual daily mean durations and number of bouts as well as the total durations and total number of bouts in Experiment 2 because individual daily means might more accurately reflect variation in the excavating activity between days. When the sample size was smaller than ten we computed exact p-values.

In order to study tool selection by the chimpanzees, we conducted the following analyses.

To investigate which physical characteristics of the tools provided by the experimenter in Experiment 1 determined if they were selected for excavating or not (“selected tools” versus “non-selected tools”), we used a Generalized Linear Model with binomial error structure and logit link function [47]. As test predictors we used the length, weight, maximum and minimum diameters of the tools provided by the experimenter. The material of the tool was not included in the model as there was complete separation for some of the levels of the factor. The covariates were successfully log transformed after visual inspection of their distributions. When examining the model stability, we found that the leverage (0.47) was slightly above the leverage threshold (0.11), indicating that there was a *potentially* high influence of some values in the data set. DFBetas revealed no problems. To establish if the model was significant, we compared the full model including all predictors with the null model only including the intercept [50] using a likelihood ratio test [49] with the test Chisq in the R function *anova*.

To determine, in Experiment 1, whether the tools the chimpanzees obtained from the natural vegetation of the island and transported to the study area to use for excavation (“selected-non-provided tools”) had similar physical characteristics to the tools they selected from those provided by the experimenter (“selected tools”), we used the two-tailed Wilcoxon rank sum test for independent samples comparing the physical characteristics (length, minimum and maximum diameter and weight) of “selected tools” versus “selected-non-provided tools”.

To determine whether chimpanzees selected excavating tools with different physical characteristics between Experiments 1 and 2 (when tools were provided versus when tools were not provided), we used the two-tailed Wilcoxon rank sum test for independent samples comparing the physical characteristics (length and weight) of “selected-non-provided” tools in Experiment 1 versus “selected-non-provided” tools in Experiment 2. Here “selected-non-provided tools” included both those the chimpanzees obtained from the natural vegetation of the island (Experiments 1 and 2) or from the fresh branches they received as enrichment in their indoor enclosure (Experiment 2), and brought to the study area to use in excavation.

When reporting the results of Wilcoxon tests, W represents the summed ranks of one of the compared groups. Data are reported as mean ± SEM and significance is established at 0.05.

## Results

### Description of excavating behaviors (Experiment 1)

**Fig 3:**
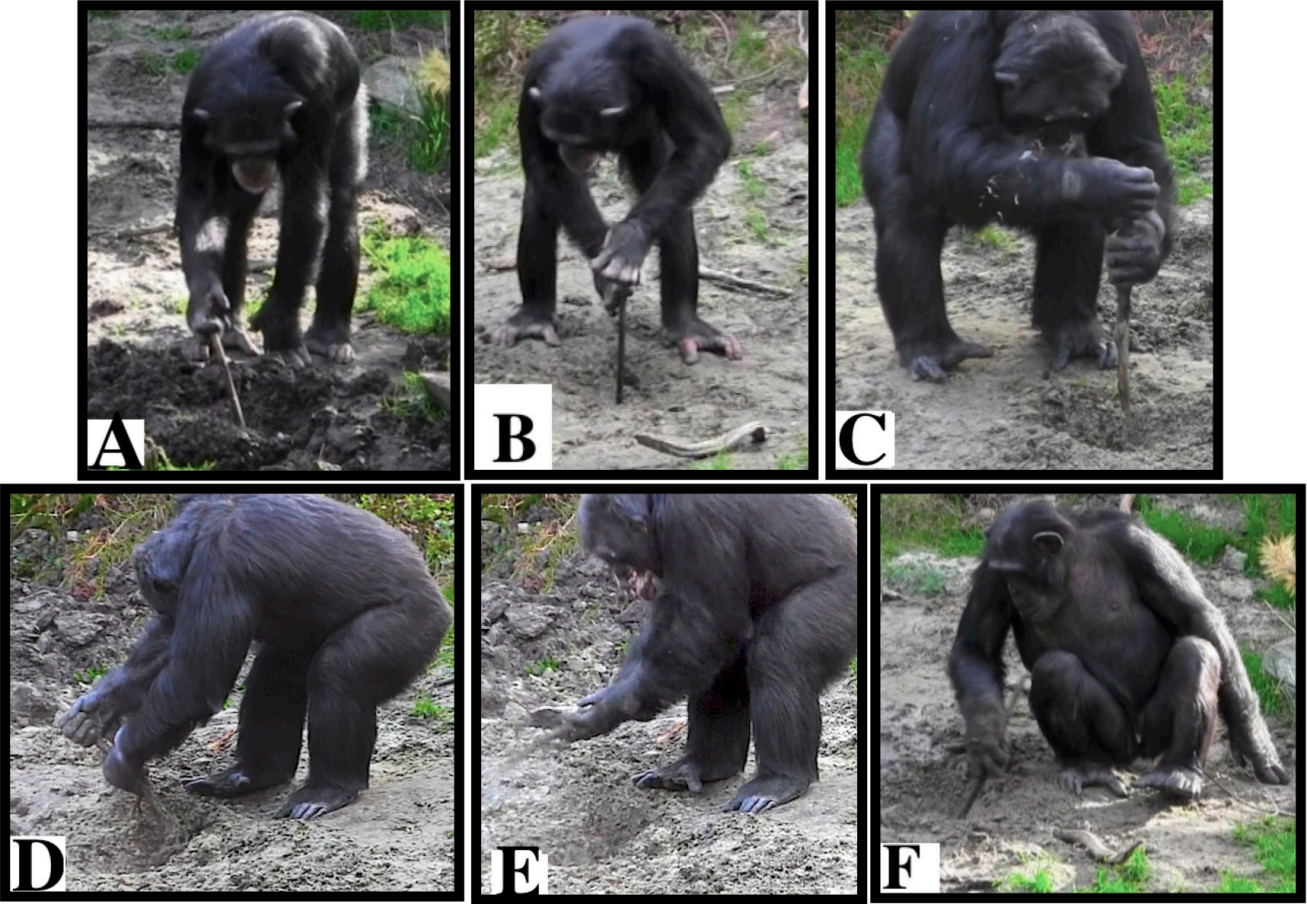
Tool use behaviors that emerged during the excavation of underground food. A) Probe, B) Perforate, C) Pound, D) Dig, E) Shovel, and F) Enlarge.

Nine out of the 10 chimpanzees were observed to excavate manually (all individuals except the infant), with seven of them (3 females and 4 males) using tools to do so. Excavating for underground foods involved six different tool use behaviors: probe, perforate, pound, dig, shovel and enlarge (S2 Table and Fig 3).

**Fig 4:**
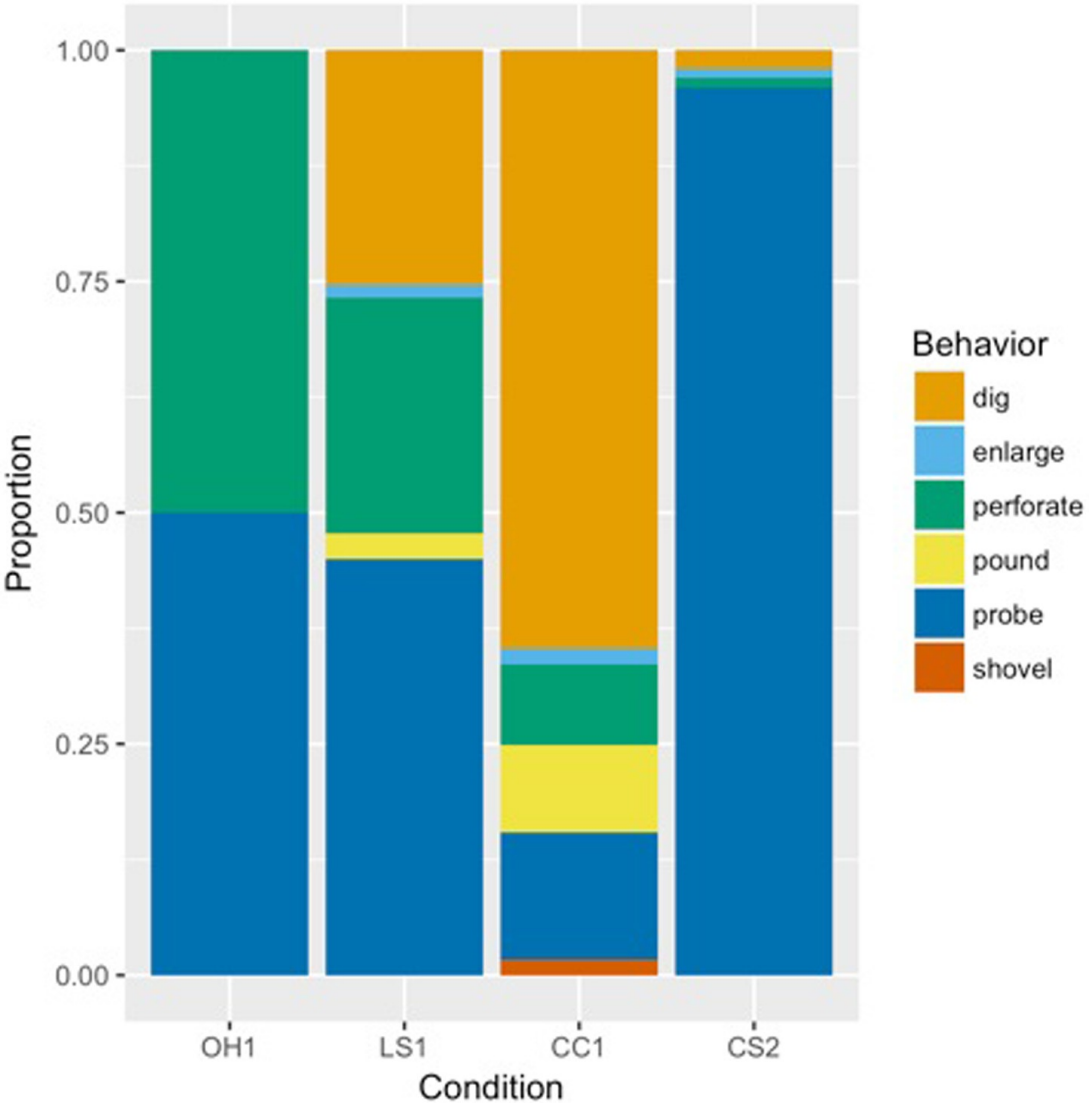
Plot of the proportions of each excavating behavior performed during each experimental condition. The abbreviations stand for *Open Hole Condition* of Experiment 1 (OH1, N = 4), *Loose Soil Condition* of Experiment 1 (LS1, N = 71), *Compacted Clay Condition* of Experiment 1 (CC1, N = 240) and *Compacted Soil Condition* of Experiment 2 (CS2, N = 81).

During the *Open Hole Condition* the chimpanzees predominantly investigated the holes visually and olfactorily. They had not been provided with tools yet, but they collected their own tools from the vegetation of the island and used them to investigate the holes by probing and perforating the natural soil at the bottom of the holes. Four tool-excavating events were observed: Julius and Junior probed on one occasion each, and Junior perforated on two occasions. During the *Loose Soil Condition* a total of 73 tool-excavating events were observed (Table 1). Probing was the excavating behavior most frequently observed (45% of the tool-excavating events), followed by digging, perforating, pounding and enlarging (Fig 4). During the *Compacted Clay Condition* 241 tool-excavating events were observed (Table 2 and Fig 4). Digging was the most frequent excavating behavior, followed by probing, pounding, perforating, shoveling, and enlarging (Fig 4).

**Table 1:** Number of events of each excavating behavior performed by each individual during the *Loose Soil Condition* of Experiment 1. In two events the individual could not be identified.

	Tool-excavating behaviors					
Individual	Dig	Enlarge	Perforate	Pound	Probe	Total
Binni	0	0	0	0	2	2
Josefine	0	0	0	0	16	16
Julius	10	0	2	1	0	13
Junior	5	0	16	1	8	30
Knerten	3	1	0	0	3	7
Miff	0	0	0	0	0	0
Tobias	0	0	0	0	3	3
Total	18	1	18	2	32	71

**Table 2:** Number of events of each excavating behavior performed by each individual during the *Compacted Clay Condition* of Experiment 1. In one of the events the individual could not be identified.

	Tool-excavating behaviors						
Individual	Dig	Enlarge	Perforate	Pound	Probe	Shovel	Total
Binni	0	0	0	0	0	0	0
Josefine	39	0	8	6	15	0	68
Julius	79	0	4	8	5	4	100
Junior	9	1	1	0	5	0	16
Knerten	3	2	0	1	2	0	8
Miff	9	0	1	0	0	0	10
Tobias	16	1	7	8	6	0	38
Total	155	4	21	23	33	4	240

### Other behaviors observed in the context of excavation (Experiments 1 and 2)

During the conditions when the holes were covered in both experiments, the chimpanzees revisited the holes and excavated the partially filled holes and the soil below the level where the fruits had been (the bottom of the hole). The chimpanzees normally dug alone, but in some cases, pairs or even trios were observed excavating the same hole, taking turns. We observed that chimpanzees shared the fruits obtained during excavation 11 times, either by giving a piece of the fruit (9 times) or the whole fruit (2 times) to a begging individual. Following Feistner and McGrew [51] sharing occurred when a chimpanzee, still interested in a defensible food item (in this case a fruit that she/he had excavated), voluntarily transferred the fruit or a piece of it to a begging individual. Following van Lawick-Goodall [52], begging occurred when an individual tried to obtain food from the possessor using persistent supplicant gestures. Three begging gestures were observed: the begging individual reached out an extended hand towards the fruit owner, got in a close face-to-face posture with the owner or gently pulled the owner’s hands. Once we saw a chimpanzee stealing a fruit (snatching and running away) from the subject who had excavated it but was distracted. We observed individuals eating fruits or pieces of fruit that were abandoned by others in the study area fifteen times. In one occasion an individual gently took a tool that another individual was using without agonistic reactions from either individual.

We observed two kinds of tool transport during Experiment 1. First, the chimpanzees collected tools on the island and transported them to the study area. Second, the chimpanzees transported tools between the holes, within and nearby the study area. Throughout the duration of this experiment the chimpanzees transported tools on 93 occasions for a total transport distance of 174.5 meters, each event ranging from 1–10 m. During Experiment 2 the chimpanzees were also observed to arrive at the study area carrying tools. The origin of the tools was either branches provided in the indoor enclosure (the zoo staff supplied fresh branches to the chimpanzees every day as part of their enrichment program) or the naturally occurring vegetation available on the island. However, the branches supplied in the indoor enclosure were not marked and therefore it was not possible to collect data on the origin of the tools the chimpanzees used during this experiment.

### Effect of soil compactness on tool use frequency (Experiment 1)

No significant effect of the differing soil compactness between conditions (*Loose Soil* and *Compacted Clay* conditions) was found on the individual tool use frequency per day when the full and reduced models (not containing test predictors) were compared (*X*^2^ = 0.455, df = 1, *p* = 0.499, See model estimates S1 Appendix).

### Excavating modalities (Experiment 1 and 2)

Eight out of nine chimpanzees (all except the adult female Dixi, S1 Table) were observed excavating manually and seven excavated with tools. All the other individuals excavated at least once in at least one experiment. Out of the seven individuals who excavated with tools in both experiments, six were the same individuals. One female excavated with tools in Experiment 1 but not in Experiment 2 and another female did the opposite (see Tables 2, 3, 4 and 5). When excavating manually the chimpanzees used various techniques, which resulted from the different locations in which the soil could be deposited relative to the chimpanzees' body (S3 Table). The chimpanzees were never observed to use both hands at the same time for excavating.

**Table 3:** Total individual bout counts and total bout durations in seconds of the manual and tool excavating bouts. The data correspond to the observations made during the *Compacted Soil Condition* of Experiment 2.

	Manual bouts		Tool bouts	
Individual	Total N bouts	Total duration (s)	Total N of bouts	Total duration (s)
Jane	36	3619	1	44
Josefine	157	10263	36	4067
Julius	142	3961	23	652
Junior	56	969	8	111
Knerten	82	2003	1	2
Miff	24	740	2	3
Tobias	69	8320	0	0
Yr	105	2882	1	22

**Table 4:** Mean daily individual bout counts ± SEM and mean daily bout durations in seconds ± SEM of manual and tool excavating bouts. The data correspond to the observations made during the *Compacted Soil Condition* of Experiment 2.

	Manual excavation bouts		Tool excavation bouts	
Individual	Mean daily N of bouts	Mean daily duration ± SEM	Mean daily N of bouts	Mean daily duration ± SEM
Jane	3.3 ± 1.0	329.0 ± 175.4	1	44.0
Josefine	4.8 ± 0.5	311.0 ± 50.7	2.3 ± 0.4	254.2 ± 103.5
Julius	3.6 ± 0.3	99.0 ± 12.4	2.1 ± 0.4	59.3 ± 12.4
Junior	3.1 ± 0.4	53.8 ± 14.8	2.7 ± 1.7	37.0 ± 29.0
Knerten	4.6 ± 0.6	111.3 ± 28.4	1	2.0
Miff	2.2 ± 0.4	67.3 ± 29.3	1	3.0
Tobias	3.5 ± 0.6	416.0 ± 144.5	0	0.0
Yr	3.6 ± 0.8	99.4 ± 25.2	1	22.0

**Table 5:** Tool dimensions. a) Tools provided to the chimpanzees during Experiment 1—those that they used as tools (Selected tools Exp 1) and those that they did not (Non-selected tools Exp 1); b) tools the chimpanzees obtained themselves from the natural vegetation of the island during Experiment 1 (Selected-non-provided tools Exp 1) and Experiment 2 (Selected-non-provided tools Exp 2). Diameters were not measured for these tools; c) Bamboo skewers (from the flags used to mark the presence of buried food) the chimpanzees used in Experiment 2 (Skewers Exp 2).

		Weight (g)		Length (cm)		Minimum diameter (cm)		Maximum diameter (cm)	
Tool types	N	Mean ± SEM	Range	Mean ± SEM	Range	Mean ± SEM	Range	Mean ± SEM	Range
Non-selected tools Exp 1	85	28.3±3.9	1.0–206.0	25.5±1.1	11.5–55.7	1.40±0.11	0.2–4.3	1.56±0.11	0.4–4.6
Selected tools Exp 1	25	58.8±9.6	1.0–162.0	44.7±1.8	28–57.5	1.38±0.15	0.3–2.9	1.68±0.17	0.4–3.2
Selected-non-provided tools Exp 1	10	52.6±19.6	9.0–189.1	53.2±6.4	24.5–81.0	1.45±0.38	0.3–3.6	2.43±0.35	0.6–4.0
Selected-non-provided tools Exp 2	42	21.8±5.7	1.0–222.0	33.7±2.7	10.4–98.0	NA	NA	NA	NA
Skewers Exp 2	44	2.3 ± 0.1	2.0–6.0	27.2±0.6	13.5–30.0	0.3	NA	0.3	NA

In the *Open Hole Condition* of Experiment 2 all the excavation bouts were manual. In three out of the 15 excavating bouts observed during this condition, soil was extracted from the hole and placed outside of the hole. In the remaining bouts no soil displacement took place. During the *Compacted Soil Condition* of Experiment 2, we recorded 671 bouts of manual excavation (90%) and 71 bouts in which tools were used to excavate (10%) (Tables 3 and 4).

The mean duration of all excavating bouts across the period of this experiment was 50.7 ± 29.8 seconds. The mean duration of all manual bouts was 48.8 ± 3.6 seconds and the mean duration of all tool use bouts was 68.1 ± 12.3 seconds (Table 3). At the individual level, all individuals excavated more often manually than using tools when the total counts of bouts from both modalities were compared (N = 8, W = 36, *p* = 0.004, Table 3). All individuals excavated longer manually than using tools when the total durations of manual and tool bouts (the sum of all bout durations of each modality performed by each individual) were compared (N = 8, W = 36, *p =* 0.004, Table 3). When individual daily mean durations of bouts were calculated and compared (Table 4), manual bouts were significantly longer than tool excavating bouts (N = 8, W = 36, *p* = 0.004). In other words, the mean time spent excavating manually per day by each individual was longer than the mean time excavating using tools per day. The individual daily mean number of bouts was higher for manual excavation than for tool excavation (N = 8, W = 36, *p* = 0.004). During this experiment, 81 tool excavating events were observed in the 71 bouts of tool excavation, most of which were classified as probing, followed by digging, enlarge and perforate (Fig 4 and S4 Table).

### Tool selection (Experiments 1 and 2)

During Experiment 1, when tools were provided by the experimenter, out of 110 provided stick tools, the chimpanzees selected 25 (23%) and used them as tools ("selected tools Exp 1" in Table 5) during the *Loose Soil* and *Compacted Clay* conditions. These tools were all from trees. We found that length was the only tool characteristic that significantly influenced the probability of a tool (among those provided by the experimenter) to be used for excavation (*X*^2^ = 51.52, df = 4, *p* < 0.001; See model estimates in S1 Appendix). The chimpanzees did not use any of the bark tools provided. During Experiment 1 the chimpanzees collected 10 sticks, a plastic tube and a blade of grass (N = 12), and transported them up to 10 m to the study area to use them as tools ("selected-non-provided tools Exp 1" in Tables 5 and S5). These tools (excluding the grass blade) did not differ from the selected tools in length (W = 156.5, *p* = 0.258), weight (W = 106.5, *p* = 0.511), maximum diameter (W = 174.5, *p* = 0.073) or minimum diameter (W = 121, *p* = 0.898). On only one occasion, in Experiment 1, a chimpanzee was observed to intentionally modify a tool by stripping the bark from a stick, peeling it down with one hand. See S5 Table for data on individual tool use.

During Experiment 2, when the chimpanzees were not provided with tools, they selected and transported 40 sticks, one long pine cone and a PVC tube ("selected-non-provided tools Exp 2" in Table 5) to the study area. These selected-non-provided tools were significantly shorter (W = 346.5, *p* = 0.012) and lighter (W = 351, *p* = 0.009) than the selected-non-provided tools in Experiment 1. In Experiment 2, the chimpanzees used 44 skewers for excavating ("skewers Exp 2" in Table 5), which were part of the flags to mark the holes in the study area, although all but one of them were used for only one excavating behavior: probing.

### Tool reuse (Experiment 1)

During Experiment 1, out of the 25 tools selected by the chimpanzees from those provided by the experimenter, 16 (64%) were reused in a total of 266 tool-excavating events throughout the experiment: 61 during the *Loose Soil Condition* and 205 during the *Compacted Clay Condition*. Six out of 12 selected-non-provided tools in Experiment 1 (50%) were also reused, in a total of 40 events: two during the *Open Hole Condition*, 11 during the *Loose Soil Condition*, and 27 during the *Compacted Clay Condition*. Fourteen of the selected and two of the selected-non-provided tools were reused on different days. See Tables 6 and S6 for data on individual tool reuse. No data on the reuse of tools were collected during Experiment 2. The addition of the tools the chimpanzees brought to the study area from the natural vegetation of the island likely influenced the frequency of tool reuse in our study, as the tools the chimpanzees selected from the ones we provided may had been reused more often without the inclusion of the new tools.

**Table 6:** Number of tools reused and number of reuse events per individual during Experiment 1.

		Selected tools		Selected-non-provided tools	
Individual	Material	N different tools reused	N of reuse events	N different tools reused	N of reuse events
Binni	Stick	2	2	0	0
Josefine	Stick	14	57	2	25
Julius	Stick	11	100	3	5
Junior	Stick	13	42	2	4
Knerten	Stick	6	14	1	1
Miff	Stick	4	10	0	0
Tobias	Stick	8	33	2	3
	Grass	0	0	1	2

## Discussion

All but one adult female chimpanzee succeeded in excavating underground food in at least one of the experiments conducted, and the majority of the chimpanzees also did so with tools (seven out of ten and seven out of nine in Experiments 1 and 2, respectively). This is an especially interesting observation, considering that the chimpanzees were naïve with regard to excavating at the onset of this study. The only other study that investigated the excavating behavior of a previously naïve great ape, reported that four out of seven bonobos at the Bonobo Hope Sanctuary (Des Moines, USA) and two out of eight at the Wuppertal Zoo (Wuppertal, Germany) used tools to excavate buried food at least once [40]. Thus, both naïve captive chimpanzees and naïve captive bonobos spontaneously used tools for excavating underground food.

From the beginning of our study, during the *Open Hole Condition* of Experiment 1, the chimpanzees used tools to probe and perforate in the context of excavation. These two behaviors were usually accompanied by careful visual and olfactory inspection of the tools after use. The same type of probing and perforating tool-inspection behavior has been observed in chimpanzees engaging in extractive foraging behaviors in the wild [53, 54], prompting researchers to suggest an exploratory function for these tools. Chimpanzees in Goualougo used puncture tools in subterranean nests to create tunnels to underground termite chambers or galleries, which are about 30 cm under the ground [55]. Sanz et al. [55] suggested that the visual and olfactory inspection of a perforating tool informs the chimpanzee if the termite nest has been accessed. Similarly, Boesch et al. [56] and Estienne et al. [54] suggested that the perforating tools chimpanzees from Loango National Park, Gabon, use in the context of extracting honey from underground hives, function to search the soil and locate the invisible nest chamber. Investigatory tool probing, a general behavior used to examine cavities, has been recorded in all long-term wild chimpanzee study sites (and thus termed a chimpanzee ‘universal’ behavior) as well as in captivity [57–60]. For example, in the specific case of aboveground honey-gathering, it is suggested that the function of probing tools is to investigate bee presence, hive access and nest solidity [53]. The first tool behavior used by five out of the seven chimpanzees who used tools to excavate in our Experiment 1 was probing. After the initial use of tools for probing and perforating by the chimpanzees in our study, other tool excavating behaviors to access the underground food emerged. We hypothesize that in any given excavation event of a sealed cavity by wild chimpanzees and hominins, investigatory probing may have been the first tool behavior used, followed by other tool-assisted excavating behaviors that allowed the extraction of the food source. Investigatory probing tools may have been used to inspect both the holes and the USO remnants left by sympatric mammals excavating USOs. Falótico et al. [34] reported that both bearded capuchins and peccaries (*Pecari tajacu*) in Serra da Capivara National Park eat the same genus of USOs and suggested that the monkeys might use the peccary excavation sites as indicators of USO location. Encountering USO excavation sites of warthogs (*Phacochoerus aethiopicus*), bushpigs (*Potamochoerus porcus*) and porcupines (*Hystrix africaeaustralis*) in the savanna woodland of Ugalla is not uncommon (Hernandez-Aguilar, unpublished data). For another underground food, Estienne et al. [61] reported that chimpanzees in Loango National Park attempted to extract honey from sites previously excavated by sympatric species such as forest elephants (*Loxodonta cyclotis)* and honey badgers (*Mellivora capensis*). Suid species, which are thought to have included USOs in their diet, are abundant in hominin fossil sites [62] and are likely to have left USO excavation sites in the ancient landscapes they shared with our ancestors.

A total of six tool behaviors (probe, perforate, pound, dig, shovel and enlarge) in the context of excavation were observed in our study. Some researchers have reported that primates exhibit more than one tool excavating behavior when foraging for underground food sources. Estienne et al. [54] described four tool excavating behaviors (referred as actions in their study) undertaken by wild chimpanzees: perforate, pound, rotate (equivalent to enlarge in our study) and lever side (no equivalent in our study). Roffman et al. [40] reported several excavating behaviors in captive bonobos including "vertical forcing", "vertical pounding", "various lever actions", "expanding the edges of the excavation" and "penetrating deep into the ground". Although a detailed ethogram was not provided, limiting the comparisons that can be made, there seems to be some overlap in the excavating behaviors used by bonobos and chimpanzees. Falótico et al. [34] described two different uses of stones by capuchin monkeys when excavating for underground food sources (USOs and arthropods). The monkeys used stones as percutors to pound and loosen the soil and as hoes to pull dirt from the digging site. Thus, chimpanzees, bonobos and capuchins all seem to exhibit pounding in the context of excavation.

When wild primates excavate natural substrates, tools may not be necessary in all contexts. In Tongo, the soil was sandy and thus easy for the chimpanzees to excavate by hand [28].

Interestingly, the way in which Bossou chimpanzees, black capuchins, and humans all obtain cassava (*Manihot esculenta*) roots is the same manual process of pulling a plant’s stem up, using only minimal superficial breaking of the soil occasionally [27, 63, 64]. The thick stem of this cultigen is a product of artificial selection by humans [64] and makes excavation unnecessary for harvesting its roots. In our study we predicted that more compact soil would elicit a higher frequency of tool use by the chimpanzees. When we assessed the effect of soil compactness in the frequency of use of tools for excavating, we found that the individual frequency of tool use per day did not differ between the *Loose Soil Condition* and the *Compacted Soil Condition* of Experiment 1, contrary to our prediction. A possible explanation for this result could be that the substrates in the two conditions did not differ enough in compactness to elicit differences in tool use frequency.

When the chimpanzees in our study were compared at the individual level regarding excavating modalities, the total number of bouts, the total bout duration, the mean daily bout duration and the mean daily number of bouts were significantly higher when excavating manually than when using tools. These differences have several possible explanations. First, tool excavation could be more effective than manual excavation in the sense that by using tools to excavate, buried food items might be obtained faster and in fewer bouts than using hands alone. Unfortunately, we were not able to test this hypothesis, as excavating effectiveness could not be measured because the moment when a food piece was obtained was not always possible to determine from video recordings. Contrary to our results, the mean time that wild chimpanzees in Loango National Park (N = 18) spent using tools to excavate underground bee nests was longer than the time they spent excavating manually [54]. This suggests that different factors, such as the difficulty in locating the underground food source and accessing it, affect the duration (and number) of the excavating bouts employed by the chimpanzees. Second, the chimpanzees dug successively in the same holes both before and after the buried food items were retrieved. The time that a hole had been previously excavated is likely to have affected the soil compactness and hole dimensions, which, in turn, is likely to affect the time a certain individual excavated. Third, environmental conditions are also likely to influence soil characteristics, specially water content, which may in turn affect how long chimpanzees need to excavate to obtain a food item (but see [34]). Future studies should test these hypotheses ideally in a savanna habitat where soil characteristics and environmental conditions may be similar to those of some habitats reconstructed for early hominins [35, 36].

Chimpanzees are known to select objects to use as tools for specific tasks based on the physical characteristics of the available objects [42, 43, 65, 66]. Chimpanzees in our Experiment 1 chose longer tools for excavating, supporting our hypothesis that they would select tools based on physical characteristics that would make them adequate for excavation. They also obtained tools of similar dimensions from (mainly) the naturally occurring vegetation of the island and transported them to the excavating site. The chimpanzees in our Experiment 1 reused some of the tools they selected in different excavating events and on different days, similar to what has been reported in studies of tool reuse by wild chimpanzees [43] and supporting our prediction that they would reuse tools. During Experiment 2, when the chimpanzees were not provided with tools on the island, they used as tools both stick tools and the thin and flimsy skewers that we used to make the hole-marking flags. Contrary to our prediction that tools selected in Experiment 2 would share similar physical characteristics with the tools selected by the chimpanzees during Experiment 1, the tools selected in Experiment 2 were shorter and lighter than those selected in Experiment 1. It is possible that as excavating tools were removed every morning from the study area by the experimenter, chimpanzees might have run out of optimal excavating tools, having to use less robust tools such as the skewers. This would explain the differences found between the selected-non-provided tools from Experiments 1 and 2. However, we do not think that the removal of the excavating tools from the study area had a strong impact on the characteristics of the excavating tools, as the island where the study took place had plenty of trees and shrubs that either drop sticks on the ground or that could be used as sources for making tools. Both are used by the chimpanzees for other tool use tasks than excavation (Hernandez-Aguilar et al., unpublished data). However, we did not measure the availability of raw material on the island and therefore can not adequately test the hypothesis that the daily removal of tools from the study area significantly affected the availability of optimal tools and led to differences in tool characteristics of the selected tools between the two experiments of our study.

The habitats of some *Australopithecus* as well as *Paranthropus* and *Homo* sites have been reconstructed as ranging from closed woodlands to shrublands, which, based on analogy with similar modern habitats, probably held an abundance of USOs [62, 67]. Fallback foods are often critical for organisms when entering new environments, especially seasonal ones [3]. Thus, it is not unlikely that USOs served as such fallback resources in the increasingly seasonal habitats occupied by hominins as the Pliocene and Pleistocene progressed. However, in such dry environments, the soil hardness may have been a significant obstacle for hungry hominins to have overcome. For example, the extreme hardness of the soil in the savanna woodland of Ugalla during the dry season may be one factor that prevents the chimpanzees from obtaining USOs during this part of the year [31]. Even sticks may not be sufficiently sturdy enough to breach densely compacted sediment under such conditions. Bearded capuchins living in another dry and seasonal environment use stones rather than plant tools in the excavation of underground food, even if they readily use sticks for tasks other than excavation [34]. Before fire was used to harden excavating sticks, plant tools may have been inefficient to use for breaking the very hard soil of arid environments during the dry season. If early hominins obtained USOs during this season, they may have obtained them either from locations where the soil was sandy or from water [28, 68], but if the soil was hard they may have required either sturdy bone or sharp stone tools for excavation. Zihlman *et al*. [69] hypothesized that together with pointed sticks, stones may have been one of the earliest tools to be used by hominins for excavating USOs but were unlikely to have been modified enough by this activity to be identifiable in the archaeological record. In addition, modern human hunter-gatherers have been observed using stones as excavating tools (reviewed in [70]). We do not contend that extant apes are “living fossil” stand-ins for our ancient ancestors. However, it seems reasonable to conjecture that at least some Pliocene and Pleistocene hominins, who–in addition to their archaeologically documented tool-making and–using capabilities (e.g., [71, 72]) had brains as large as and larger than those of *Pan* [73] and hands more manipulative than those of *Pan* (e.g., [74, 75, 76, 77]), would have been capable of using simple implements to harvest USOs.

Captivity produces uncommon environmental and social conditions for animals, such as increased contact between individuals and lack of predation [78]. We observed several social behaviors in the context of underground food excavation in our study. The chimpanzees saw each other excavating, and sometimes excavated socially (even the same holes) and shared the excavated food. Thus, in the scenario of using USOs as fallback foods, similar social behaviors would have provided adaptive benefits for early hominin individuals excavating in groups, such as protection from predators and opportunities for tool learning by immature individuals. The hypothesized abundance of USOs in hominin habitats would have prevented intergroup food competition. Both wild bearded capuchins and chimpanzees have been reported to reuse USO excavating sites [31, 34] and thus such sites may provide excavation opportunities for inexperienced individuals [79], especially if tools left at the excavation sites would be reused as it was the case in our study.

Our results demonstrate, for the first time, that excavating-naïve chimpanzees are able to spontaneously use tools in order to excavate artificially buried food. Further, the majority of individuals in our study succeeded in excavating with tools. Interestingly, we found that other behaviors besides digging were involved in the tool-assisted excavation of underground food: probing, perforating, pounding, shoveling and enlarging. As such, the excavation of artificially buried food (and presumably the excavation of USOs) does not involve a single tool behavior but rather a repertoire. This would suggest that USO excavation by wild chimpanzees may be similar in complexity to other underground food source extractive tasks such as foraging for underground honey bee nests [54] or termites [55]. In the context of underground food excavation, the chimpanzees in our study selected tools for the task, reused some tools more frequently than others and transported tools. These behaviors have already been reported for chimpanzees in other contexts, considered complex, such as foraging for termites [55], honey [56] and nut kernels [43]. Here we show that they also occurred in a context analogous to the excavation of wild USOs, further suggesting that USO excavation by wild chimpanzees may be a complex task. However, captive primates may exhibit a higher frequency and diversity of tools use than their wild counterparts (captivity bias effect, [69]) and thus direct observations of wild chimpanzees excavating USOs are needed to confirm this hypothesis. Our results highlight the importance of conducting experiments with captive primate populations to enable us to better understand the behavior of their non-habituated, unobservable wild conspecifics that can only be indirectly studied using archaeological methods (sensu [80]) and to explore the implications of such behaviors for human evolution.

## Supporting information

S1 Table Demographic data of the individuals included in the experiments. (DOCX)

S2 Table Ethogram of tool use behaviors involved in excavation. (DOCX)

S3 Table Ethogram of manual techniques observed during Experiment 2. (DOCX)

S4 Table Number of tool-excavation events performed by each individual during the *Compacted Soil Condition* of Experiment 2. (DOCX)

S5 Table Dimensions of the selected and selected-non-provided tools used per individual during Experiment 1. (DOCX)

S6 Table Number of times each tool was reused and individual count of reuse events. Eight reuse events are not included in the table because the individuals that reused the tools could not be identified.
(DOCX)

S1 Appendix Model structures and estimates. (DOCX)
