# Supplementary material for: Chimpanzee extractive foraging with excavating tools: Experimental modeling of the origins of human technology
Source: PLoS One. 2019 May 15;14(5):e0215644. doi: 10.1371/journal.pone.0215644 (PMC6519788; doi:10.1371/journal.pone.0215644)
Supplement: S1 Table — (DOCX) [file pone.0215644.s001.docx]

| **Name** | **Sex** | **Year of birth** | **Age class** | **Group** | **Origin** | **Human reared** | **Parents** | **Offspring** |
| --- | --- | --- | --- | --- | --- | --- | --- | --- |
| Binni^1^ | F | 1974 | Adult | 1 |  | Yes |  | Junior |
| Dixi | F | 1977 | Adult | 2 | Munich Zoo | No | - | Jane/Tobias |
| Julius | M | 1979 | Adult | 1 | Kristiansand Zoo | Yes | - | Junior/Yr |
| Josefine | F | 1983 | Adult | 2 | Öland Zoo | No | - | - |
| Miff | F | 1987 | Adult | 1 | Copenhagen Zoo | No | - | Knerten |
| Tobias | M | 1994 | Adult | 2 | Kristiansand Zoo | No | Dixi | - |
| Jane | F | 1999 | Adult | 2 | Kristiansand Zoo | No | Dixi | Yr |
| Knerten | M | 2000 | Adult | 1 | Kristiansand Zoo | No | Miff | - |
| Junior | M | 2003 | Adult | 1 | Kristiansand Zoo | No | Binni/Julius | - |
| Yr | F | 2011 | Infant/juvenile | 2 | Kristiansand Zoo | No | Jane/Julius | - |

1. Binni was only present in Experiment 1
