# Supplementary material for: Chimpanzee extractive foraging with excavating tools: Experimental modeling of the origins of human technology
Source: PLoS One. 2019 May 15;14(5):e0215644. doi: 10.1371/journal.pone.0215644 (PMC6519788; doi:10.1371/journal.pone.0215644)
Supplement: S2 Table — (DOCX) [file pone.0215644.s002.docx]

| **Behaviour** | **Description** |
| --- | --- |
| Probe | A chimpanzee holds one end of a tool and places the other end in a hole without using his/her body weight (generally while holding the tool with a precision grip [1] but not necessarily). The hole can be open, completely or partially excavated. The tool is then withdrawn and the inserted end may or may not be visually and olfactorily inspected. |
| Perforate | A chimpanzee inserts a tool into the ground and applies force pushing the end of the tool into the ground with both hands or a hand and a foot, or one or both feet. Power grip is generally (but not necessarily) used if one of the hands holds the midsection of the tool. The tool is then retrieved and the end that went into the ground may or may not be visually and olfactorily inspected. |
| Pound | A chimpanzee holds a tool with one or both hands and with powerful back and forth movements of the tool, hits the ground repeatedly. |
| Dig | A chimpanzee holds a tool with both or one hand and inserts it into the ground at approximately arm length. Then, while pressing the tool in the ground, moves it powerfully inward towards him/herself – once or repeatedly. |
| Shovel | A chimpanzee holds the midsection of the tool with one hand and with the other hand, or the opposite leg, holds the upper end of the tool. Then she/he inserts the lower end of the tool into the ground and forces it in until about half of the tool had penetrated. The chimpanzee then withdraws the tool outward, which results in the removed materials deposited outside of the excavated area. |
| Enlarge | A chimpanzee inserts a tool into an open hole and with sweeping, circular motions of the tool widens the opening. The tool stays in contact with the ground while rotating. |

1. Jones-Engel LE, Bard KA. Precision grips in young chimpanzees. American Journal of Primatology. 1996;39:1-15.
