# Supplementary material for: Chimpanzee extractive foraging with excavating tools: Experimental modeling of the origins of human technology
Source: PLoS One. 2019 May 15;14(5):e0215644. doi: 10.1371/journal.pone.0215644 (PMC6519788; doi:10.1371/journal.pone.0215644)
Supplement: S3 Table — (DOCX) [file pone.0215644.s003.docx]

| **Technique** | **Description** |
| --- | --- |
| One sided | A hand with the palm facing towards the body and the fingers pointing down moves first downwards, grasping soil, and then upwards and to the same side of the body as the working hand, releasing the dirt. |
| Cross sided | A hand with the palm facing towards the body and the fingers pointing down moves first downwards, grasping soil, and then upwards, crossing the arm in front or behind the other arm and releasing the dirt. |
| Frontal | The hand is inserted in the hole, with its back towards the body and fingers extended. The hand is moved first downwards, grasping the soil, and then front and upwards, pushing the soil out of the hole at the furthest point relative to the body. |
| No soil  displacement | With the palm facing towards the body one finger is used to poke inside the soil and moved towards the body repeatedly as if scratching. When it is pulled out it is inspected both visually and olfactorily. Alternatively, four fingers are inserted in the soil and moved towards the body repeatedly. No extraction of soil occurs and the chimpanzee leaves the loosened soil inside the hole. |
