# Supplementary material for: Chimpanzee extractive foraging with excavating tools: Experimental modeling of the origins of human technology
Source: PLoS One. 2019 May 15;14(5):e0215644. doi: 10.1371/journal.pone.0215644 (PMC6519788; doi:10.1371/journal.pone.0215644)
Supplement: S4 Table — (DOCX) [file pone.0215644.s004.docx]

|  |  | **Tool excavating behaviours** | | | | |
| --- | --- | --- | --- | --- | --- | --- |
| **Individual** | **Material** | **Probe** | **Perforate** | **Enlarge** | **Dig** | **Total** |
| Jane | skewer | 1 | 0 | 0 | 0 | 1 |
| Josefine | skewer | 26 | 0 | 0 | 0 | 26 |
| Josefine | stick | 13 | 0 | 0 | 0 | 13 |
| Julius | skewer | 15 | 0 | 1 | 2 | 18 |
| Julius | stick | 3 | 0 | 0 | 0 | 3 |
| Junior | pinecone | 1 | 0 | 0 | 0 | 1 |
| Junior | skewer | 3 | 0 | 0 | 0 | 3 |
| Junior | stick | 11 | 1 | 0 | 0 | 12 |
| Knerten | skewer | 1 | 0 | 0 | 0 | 1 |
| Miff | skewer | 2 | 0 | 0 | 0 | 2 |
| Yr | skewer | 1 | 0 | 0 | 0 | 1 |
| Total |  | 77 | 1 | 1 | 2 | 81 |
