# Supplementary material for: Chimpanzee extractive foraging with excavating tools: Experimental modeling of the origins of human technology
Source: PLoS One. 2019 May 15;14(5):e0215644. doi: 10.1371/journal.pone.0215644 (PMC6519788; doi:10.1371/journal.pone.0215644)
Supplement: S5 Table — (DOCX) [file pone.0215644.s005.docx]

|  |  | **Selected tools** | | | **Selected-non-provided tools** | | |
| --- | --- | --- | --- | --- | --- | --- | --- |
| **Individual** | **Material** | **N tools used** | **Mean weight (g)  ± SEM** | **Mean length (cm)  ± SEM** | **N tools used** | **Mean weight (g)  ± SEM** | **Mean length (cm)  ± SEM** |
| Binni | stick | 2 | 108 ± 4 | 45 ± 10.5 |  |  |  |
| Josefine | stick | 16 | 66.7 ±12.3 | 44.6 ± 2.2 | 2 | 20.5 ± 0.5 | 55.8 ± 7.8 |
| Julius | stick | 14 | 74.4 ± 12.1 | 49.8 ± 1.7 | 6 | 63.2± 30.5 | 39.5± 5.2 |
| Junior | stick | 13 | 66.9 ± 11.6 | 47.9 ± 2 | 4 | 36.8 ± 20.5 | 73.4 ± 3.6 |
| Knerten | stick | 6 | 75.7 ± 10.4 | 52.4 ± 1.7 | 1 | 19 | 28,6 |
| Miff | stick | 4 | 80.8 ±12.8 | 51.8 ±2.1 |  |  |  |
| Tobias | stick | 10 | 53.6 ± 11.7 | 42.9 ± 3.2 | 2 | 20.5 ± 0.5 | 55.8 ± 7.8 |
| Tobias | grass |  |  |  | 1 | 8 | 17 |
| Tobias | plastic |  |  |  | 1 | 90 | 34 |
