# Supplementary material for: Chimpanzee extractive foraging with excavating tools: Experimental modeling of the origins of human technology
Source: PLoS One. 2019 May 15;14(5):e0215644. doi: 10.1371/journal.pone.0215644 (PMC6519788; doi:10.1371/journal.pone.0215644)
Supplement: S6 Table — Eight reuse events are not included in the table because the individuals that reused the tools could not be identified. (DOCX) [file pone.0215644.s006.docx]

| **Individual** | **Provided tools (ID)** | | | | | | | | | | | | | | | | **Selected non-provided tools (ID)** | | | | | | **Individual**  **N of tool reuse events** |
| --- | --- | --- | --- | --- | --- | --- | --- | --- | --- | --- | --- | --- | --- | --- | --- | --- | --- | --- | --- | --- | --- | --- | --- |
|  | 100 | 101 | 102 | 103 | 104 | 107 | 108 | 110 | 65 | 69 | 78 | 93 | 94 | 95 | 96 | 97 | A | C | D | F | G | I |  |
| Binni | 0 | 0 | 1 | 0 | 0 | 0 | 0 | 0 | 0 | 0 | 1 | 0 | 0 | 0 | 0 | 0 | 0 | 0 | 0 | 0 | 0 | 0 | 2 |
| Josefine | 6 | 1 | 18 | 6 | 4 | 1 | 11 | 3 | 1 | 1 | 1 | 0 | 1 | 0 | 1 | 2 | 0 | 11 | 14 | 0 | 0 | 0 | 82 |
| Julius | 0 | 7 | 21 | 21 | 6 | 2 | 31 | 2 | 0 | 0 | 1 | 2 | 0 | 1 | 6 | 0 | 0 | 1 | 0 | 2 | 0 | 2 | 105 |
| Junior | 1 | 4 | 4 | 1 | 4 | 7 | 6 | 5 | 1 | 0 | 2 | 2 | 0 | 1 | 4 | 0 | 2 | 0 | 2 | 0 | 0 | 0 | 46 |
| Knerten | 0 | 1 | 2 | 4 | 0 | 0 | 3 | 2 | 0 | 0 | 0 | 2 | 0 | 0 | 0 | 0 | 0 | 0 | 0 | 1 | 0 | 0 | 15 |
| Miff | 0 | 0 | 1 | 1 | 1 | 0 | 7 | 0 | 0 | 0 | 0 | 0 | 0 | 0 | 0 | 0 | 0 | 0 | 0 | 0 | 0 | 0 | 10 |
| Tobias | 1 | 0 | 6 | 0 | 0 | 0 | 16 | 1 | 0 | 3 | 1 | 0 | 1 | 0 | 4 | 0 | 0 | 2 | 1 | 0 | 2 | 0 | 38 |
| **N of uses  of each tool** | 8 | 13 | 53 | 33 | 15 | 10 | 74 | 13 | 2 | 4 | 6 | 6 | 2 | 2 | 15 | 2 | 2 | 14 | 17 | 3 | 2 | 2 | 298 |

* The total number of individual tool reuse events is lower here (298) than the reported in the text (306) because there where 10 times when the individual could not be identified during reuse events.
