# Supplementary material for: Chimpanzee extractive foraging with excavating tools: Experimental modeling of the origins of human technology
Source: PLoS One. 2019 May 15;14(5):e0215644. doi: 10.1371/journal.pone.0215644 (PMC6519788; doi:10.1371/journal.pone.0215644)
Supplement: S1 Appendix — (DOCX) [file pone.0215644.s007.docx]

Notes:

- P values calculated using drop1()
- Intercept statistics are not shown for having limited interpretation

Model structure:

glm(Tool_used~LogLength+ LogWeigth+ LogMaxDiameter+ LogMinDiameter, family= binomial)

|  | Estimate | SE | df | X^2^ | P |
| --- | --- | --- | --- | --- | --- |
| Intercept | -19.18 | 6.16 |  |  |  |
| LogLength | 4.83 | 1.46 | 1 | 15.31 | <0.001 |
| LogWeight | 0.624 | 0.75 | 1 | 0.67 | 0.41 |
| LogMaxDiameter | -0.123 | 2.35 | 1 | 0.003 | 0.96 |
| LogMinDiameter | -0.246 | 2.08 | 1 | 0.01 | 0.91 |

Model structure:

glmer.nb(Tool_events_day~Condition+Sex+(1+Condition.Code|Individual), family = poisson)

|  | Estimate | SE | df | X^2^ | P |
| --- | --- | --- | --- | --- | --- |
| Intercept | 1.15 | 0.53 |  |  |  |
| Loose soil condition | -0.27 | 0.39 | 1 | 0.45 | 0.50 |
| Sex | 0.624 | 0.75 | 1 | 0.96 | 0.33 |
